# Odour learning concentration influences concentration range of conditioned response in newborn rabbits

**DOI:** 10.1038/s41598-025-28326-6

**Published:** 2025-12-29

**Authors:** Gérard Coureaud, Marie-Sabelle Hjeij, Jeanne Serrano, Marc Thévenet, Samuel Garcia, Jean-Marie Heydel, Patricia Duchamp-Viret

**Affiliations:** 1 Lyon Neuroscience Research Center, CNRS UMR 5292, INSERM U 1028, Université Claude Bernard Lyon 1, Université Jean Monnet, Centre Hospitalier Le Vinatier - Bâtiment Neurocampus, 40 Avenue du Doyen Lepine, Bron, F-69500 France; 2 Laboratoire Interdisciplinaire Carnot de Bourgogne ICB UMR 6303, Université Bourgogne Europe, CNRS, 7 bvd Jeanne d’Arc, Dijon, F-21000 France

**Keywords:** Newborn, Pheromone, Odour learning, Concentration, Perception, Rabbit, Behavioural ecology, Sensory processing

## Abstract

Newborn mammals must adapt to the chemically complex environment by detecting and prioritizing relevant stimuli. In rabbits, the mammary pheromone (MP) emitted by lactating females triggers a typical behaviour in newborns, helping them to locate the nipples, suck and survive. The MP also promotes very rapid learning of other odorants by associative conditioning. In this study, MP-induced learning was used to investigate the neonatal detection and recognition abilities of two odorants very different in volatility, ethyl isobutyrate and ethyl maltol, across concentrations ranging from 10^− 5^ to 10^− 25^ g/ml. The results show, firstly, that the odorants could be learned even at very low concentrations; and secondly, that a process of generalisation of the odorant quality was effective after learning over a wide range of concentrations. However, the degree of generalisation depended on the concentration at which the odorants had been learned, with quality and intensity becoming closely interdependent for very low concentrations of learning. Taken together, these data highlight the remarkable adaptability of the olfactory perceptual and cognitive systems of newborn rabbits, enabling them not only to rapidly learn new odorants, but also to attribute qualities to them that depend on the quality perceived at the learning concentration.

**Supplementary Information:**

The online version contains supplementary material available at 10.1038/s41598-025-28326-6.

## Introduction

Perception occurs when animals create brain representations of the external world. It is critical in helping organisms make important choices among the many, varied and ever-changing sensory stimuli in the environment. This requires animals to discriminate between stimuli according to their nature but also their intensity, variations that can call for perceptual discrimination or, on the contrary, generalisation^1,2^. Thus, the power and adaptive capacity of sensory receptors, as well as the depth of neurophysiological processing of sensory inputs determine the way in which organisms interact with sensory stimuli. This shapes the perception of their own world (i.e., their Umwelt)^3^. Much remains to be discovered about the abilities of animals to adapt to changing environments as well as the cognitive and physiological limits underlying these adaptations, particularly early in life.

Animals are regularly confronted with ecological situations that push their perceptual channels beyond their routine^4^. This can involve extreme sensitivity to odours in order to cope with critical situations such as, in adult organisms, finding food, avoiding predators, choosing a mate, caring for offspring^5–9^; for example, adult male insects can detect odour molecules at remarkably low concentrations to track the pheromone plumes emitted by females^10,11^. Importantly, olfaction is also a major sensory feature of young organisms, contributing to the adaptation of individuals to the environment early in ontogeny^12^. This raises the question of the extent to which organisms have effective and sufficiently efficient olfactory detection abilities at birth, and how these performances can be modulated by learning, when the neonatal olfactory system and brain are still partially immature. Here, we tested these research questions in an animal model that has become very relevant in recent decades for assessing both predisposed and learned perinatal odour perception: the newborn rabbit.

In mammals, odour perception begins *in utero* and is vital from birth when newborns must immediately adapt to the external (aerial) environment^12–14^. Neonatal responsiveness to odours may result from predisposition to respond to spontaneously significant biological stimuli, and/or from learning about stimuli that were initially devoid of meaning^15–17^. The rabbit model illustrates these two mechanisms. Indeed, newborn rabbits are remarkably quick to locate their mother’s nipples during the single and very brief (< 5 min) daily exposure to the mother in the nest, even though they are initially blind and deaf. This is due to their perception of a monomolecular volatile component naturally emitted by lactating female rabbits in their milk, known as the mammary pheromone (MP, 2-methylbut-2-enal)^18–22^. The MP triggers the typical orocephalic nipple-search behaviour in newborns, without any learning beforehand^19,23^. The MP also acts as a potent reinforcer, i.e. an unconditioned stimulus (US), which can rapidly confer biological significance to a new odorant/mixture of odorants (conditioned stimulus, CS) that is initially inactive (i.e., not spontaneously active in behaviour). Twenty-four hours after a single exposure to CS + US for a very brief presentation period (5 min) the CS alone is sufficient to trigger the typical sucking-related behaviour in newborn rabbits^24–28^.

It has recently been shown that a single episode of MP-induced odour learning is sufficient to increase the strength of electrophysiological responses to the CS in the olfactory mucosa of rabbit pups, and their perceptual sensitivity to the CS^29^. This was the first evidence of the process of “induction” in neonates, i.e., when odour conditioning induces plasticity not only in the brain but also in the periphery of the olfactory system^30–35^. Thus, whereas newborn rabbits could not perceive a new odorant at 10^− 17^ g/ml because this concentration was below their spontaneous detection threshold for that odorant, they could respond to it at 10^− 17^ g/ml 24 h after learning the odorant by pairing with the MP; this was observed when both stimuli were used at 10^−5^ g/ml during conditioning. Therefore, MP-induced odour learning can not only confer biological value to a new odorant, but also improve the newborn’s sensitivity to that odorant by expanding their detection threshold to lower concentrations^29^.

In the present study, we aimed to confirm and extend these recent observations while examining in depth how the processes of generalisation and discrimination interact in neonatal odour detection. The experiments were conducted in 2- to 4-day old rabbit pups, using two odorants known to be learnable by newborn rabbits^26,27,29^. These odorants were deliberately chosen for their contrasting volatility properties - odorant A (ethyl isobutyrate) is highly volatile, whereas odorant B (ethyl maltol) is low in volatility - in order to reflect the natural environment, which contains odours that vary widely in volatility. First, we conditioned rabbit pups to odorant A or B, with MP concentration maintained at a constant level (10^− 5^ g/ml) while the CS concentration varied over a wide range, i.e., from 10^− 3^ to 10^− 24^ g/ml. The hypothesis was that MP could promote the learning of CS even at very low concentrations during the conditioning, with a certain limit (to be determined). Second, the responsiveness of the pups after MP-induced learning was systematically assessed not only by testing at the CS concentration to which the pups had been conditioned, but also by presenting them with a wide range of CS concentrations, including levels higher and lower than the initial CS concentration. Our hypothesis was that the concentration range within which newborn rabbits respond to CS *after* conditioning would vary according to the concentration of CS *during* conditioning, i.e., that memory may depend on the quality perceived and acquired during conditioning.

## Results

### Pup responsiveness to conditioned concentrations

To assess the concentration range over which rabbit pups can be conditioned to a new odorant by pairing it with MP 10^− 5^ g/ml, 176 and 153 pups were conditioned on day 1, 2 or 3 to odorant A from 10^− 5^ to 10^− 23^ g/ml (9 groups, one per concentration) and odorant B (8 groups, one per concentration) from 10^− 5^ to 10^− 22^ g/ml, respectively, before being tested to the conditioned stimulus 24 h after the conditioning episode. Analysis of the results then consisted of examining the responsiveness displayed to the concentration at which the CS had been learned.

**Fig. 1:**
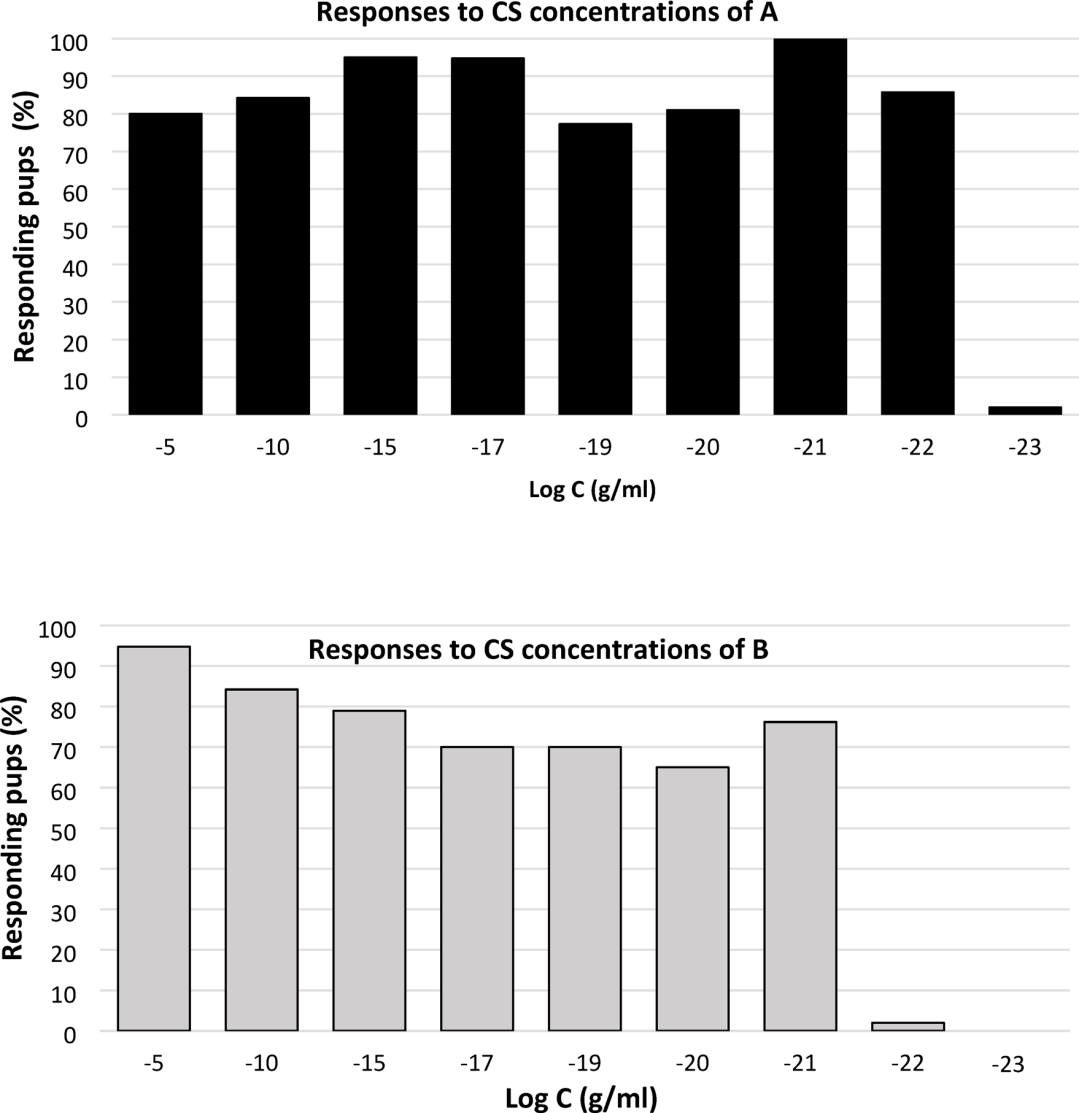
Proportions of 2- to 4-day-old rabbit pups responding in an oral activation test to (a) odorant A (ethyl isobutyrate, *n* = 176 pups) and (b) odorant B (ethyl maltol, *n* = 153 pups) at the concentration at which each stimulus has been conditioned by pairing with the mammary pheromone 24 h before, i.e. 10^− 5^ to 10^− 23^ g/ml for A, and 10^− 5^ to 10^− 22^ g/ml for B.

For odorant A, the pups highly and similarly responded to the CS when its learning concentration was between 10^− 5^ and 10^− 22^ g/ml (77–100%; χ² = 9.1; df = 7, *p* = 0.24; all statistical results are detailed in supplementary files, only the main results are presented in the text). At these concentration levels, their responsiveness was as high as to the MP itself (95–100% response to MP; 2 × 2 comparisons between learning concentrations of A vs. MP: χ² < 0.5, *p* > 0.24). However, pups did not respond to odorant A after conditioning at 10^− 23^ g/ml (2 × 2 comparisons with all other concentrations: χ² > 20.49, *p* < 0.0001), whereas they responded strongly to MP (100%; not illustrated here) (Fig. 1A).

For odorant B, the pups highly and similarly responded to the CS when its concentration of learning was between 10^− 5^ and 10^− 21^ g/ml (65–95%; χ² = 6.6, ddl = 6, *p* = 0.35). Again, this responsiveness was as high as to the MP itself (89–100%; 2 × 2 comparisons between learning concentrations of B vs. MP: χ² < 3.2, *p* > 0.05). However, their responsiveness to odorant B was null at 10^− 22^ g/ml (2 × 2 comparisons with all other concentrations: χ² > 15.51, *p* < 0.0001) whereas the pups responded to MP (100%; not shown here) (Fig. 1B).

In addition, we tested whether newborn rabbits can learn the odorants A or B at concentration levels higher than 10^− 5^ g/ml, i.e., at 10^− 3^ and 10^− 2^ g/ml. A prerequisite was to determine whether the MP could be detected in such mixtures. Therefore, 10 naive 2-day-old pups were tested for their oral responsiveness to two mixtures containing MP at 10^− 5^ g/ml and odorant A at 10^− 2^ or 10^− 3^ g/ml. Nine other naive 2-day-old pups were tested for their oral responsiveness to 2 mixtures containing MP at 10^− 5^ g/ml and odorant B at 10^− 2^ or 10^− 3^ g/ml. For odorant A, no pups responded to the mixtures, suggesting that the MP was masked by odorant A, making learning impossible at these concentrations. Indeed, when 5 and 9 pups were exposed to MP + A 10^− 2^ or MP + A 10^− 3^ g/ml, respectively, none of them responded to odorant A the following day (while all responded to the MP). In contrast, 90% and 100% of pups responded to the MP mixed with B at 10^− 2^ and 10^− 3^ g/ml, respectively, demonstrating that MP was perceived in these mixtures and, thus, that B could theoretically be learned. However, when 5 and 10 additional pups were exposed to MP + B 10^− 2^ or MP + B 10^− 3^ g/ml, respectively, only one pup conditioned with B at 10^− 3^ responded to B on day 3 (while all responded to MP) demonstrating that odorant B cannot be learned efficiently at these concentrations.

In summary, this first set of results showed that, under our conditions and with a single concentration of MP, newborn rabbits could learn odorants A or B over a wide range of concentrations (of 10^17^ and 10^16^ log-units, respectively). This range included very low concentrations (< 10^− 20^ g/ml for A and B; with certain limits: no learning occurred below 10^− 21^ g/ml for B and below 10^− 22^ g/ml for A) but excluded very high concentrations (≥ 10^− 3^ g/ml for both odorants).

### Pup responsiveness to different CS concentrations as a function of conditioning concentration

To determine how MP-induced conditioning gave rise to a qualitative generalization and/or an increase in sensitivity depending on the learning concentration, the pups conditioned above to odorants A or to B were also tested on concentrations other than the learning ones. To this end, each group of pups was divided into two subgroups tested with 5 or 6 concentrations of CS (from 10^− 5^ to 10^− 25^ g/ml and from 10^− 5^ to 10^− 22^ g/ml for A and B, respectively) systematically including concentrations expected to be below the detection/recognition threshold, then with the learning concentration, and finally with the MP as a control. Analysis of the results consisted of examining the responsiveness of the pups within and between the subgroups.

For odorants A and B, whatever the concentration of learning, the distribution of responsiveness over the ranges of concentrations differed according to the tested concentrations (global comparisons for A and B: *p* < 0.043 for A and *p* < 0.002 for B - with rare exceptions for CS B^− 17^ and B^− 19^, *p* = 0.1 -; detailed statistics are provided in supplemental materials). Below, we detail the pups’ responsiveness when it was statistically significant (all comparisons, including these, are presented in the supplementary materials).

**Fig. 2:**
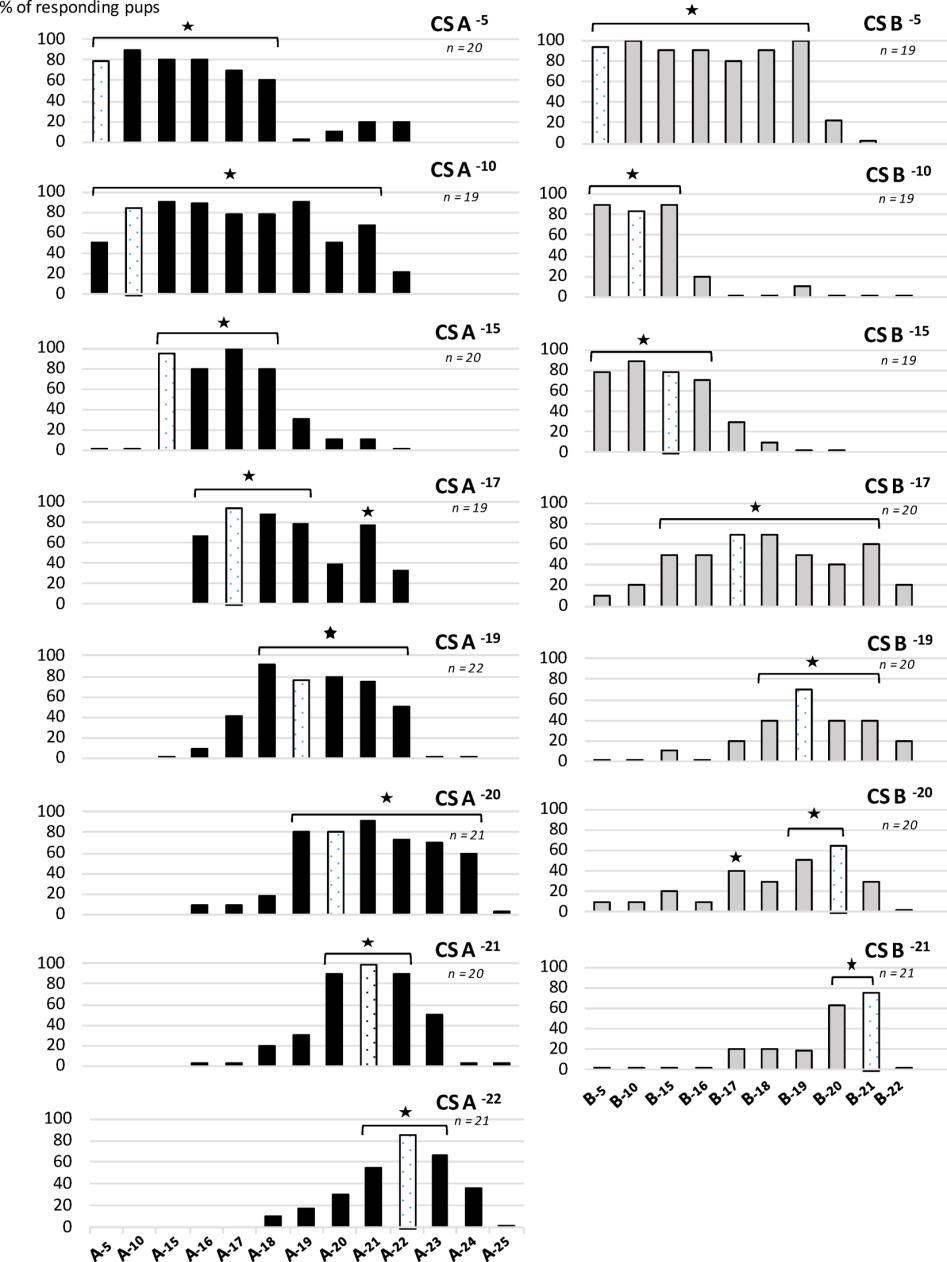
Proportions of 2- to 4-day-old rabbit pups responding in an oral activation test to the conditioned odorant A (ethyl isobutyrate, black bars, *n* = 162 pups) or odorant B (ethyl maltol, grey bars, *n* = 138 pups) 24 h after MP-induced conditioning to the conditioned stimulus (CS) at concentrations varying from 10^− 5^ to 10^− 22^ g/ml for A, and from 10^− 5^ to 10^− 21^ g/ml for B. For each learning concentration of CS, i.e., each graph, two distinct groups of pups were tested at various levels of concentrations during oral testing. In each graph, responsiveness to the CS concentration used at conditioning is marked by a white bar and, significant response rates between the highest and lowest levels of responsiveness are marked with a star (detailed statistical comparisons are provided in Supplementary Materials).

For odorant A (Fig. 2, left), conditioning at 10^− 5^ g/ml was followed by high and constant responsiveness to the stimulus (> 60%) from 10^− 5^ to 10^− 18^ g/ml. Then, a drastic drop was observed at 10^− 19^ g/ml and responsiveness remained very weak (≤ 20%) at the lowest tested concentrations (10^− 20, −21, −22^ g/ml). After conditioning at 10^− 10^ g/ml, only 50% of the pups responded at the highest concentration (10^− 5^ g/ml) while 67–90% responded over a range extending from 10^− 10^ to 10^− 21^ g/ml; this was one of the widest ranges of responsiveness observed in our results despite a transient slight decrease at 10^− 20^ g/ml. The responsiveness then severely dropped at 10^− 22^ g/ml. After conditioning at 10^− 15^ and 10^− 17^ g/ml, pups completely ceased to respond to the highest concentrations, i.e., from 10^− 5^ to 10^− 10^ and 10^− 5^ to 10^− 15^ g/ml, respectively. The range of active concentrations became limited to 10^− 15^ to 10^− 18^ g/ml (CS A 10^− 15^ g/ml: ≥ 80%) and 10^− 16^ to 10^− 21^ g/ml (CS A 10^− 17^ g/ml: ≥ 67%), with, for the latter, a relative drop at 10^− 20^ g/ml (40%), whereas 10^− 21^ induced 78% responsiveness. After conditioning at 10^− 19^ g/ml, the highest responsiveness was observed between 10^− 18^ and 10^− 21^ g/ml (≥ 75%), while responsiveness was intermediate at 10^− 17^ and 10^− 22^ g/ml (42–50%), very weak or null for 10^− 16^ and above, and 10^− 23^ g/ml and below. For 10^− 20^ g/ml of learning concentration, high responsiveness was observed from 10^− 19^ to 10^− 24^ (60–91%), but not significantly above or below these concentrations (< 20%). After conditioning at 10^− 21^ and 10^− 22^ g/ml, the range of concentrations triggering the highest responsiveness became narrower and centred on the learning concentration, i.e., from 10^− 20^ to 10^− 22^ g/ml (90–100%) and from 10^− 21^ to 10^− 23^ g/ml (55–86%) respectively. Finally, after conditioning at 10^− 23^ g/ml (not shown in Fig. 2), no pups responded to A 10^− 23^ g/ml (Fig. 1A) or to any of the other concentrations tested (from 10^− 18^ to 10^− 25^ g/ml), attesting that the learning concentration threshold for odorant A is ≤ 10^− 22^ and > 10^− 23^ g/ml.

For odorant B, we observed the same global trend as for odorant A in pup responsiveness, but with a different dynamic. After conditioning at 10^− 5^ g/ml, responsiveness was high (≥ 80%) over a wide range of concentrations, i.e., from 10^− 5^ to 10^− 19^ g/ml, then severely decreased at 10^− 20^ g/ml (22%) and completely ceased below this concentration. After conditioning at 10^− 10^ and 10^− 15^ g/ml, pups responded highly over a narrower range, anchored along the highest concentrations, i.e., from 10^− 5^ to 10^− 15^ g/ml (84–89%) and 10^− 5^ to 10^− 16^ g/ml (70–89%), respectively, while responsiveness was weak at lower concentrations (≤ 30%). After conditioning at 10^− 17^ g/ml, the range of pup responsiveness became again considerably broader: pups responded over a range of concentrations extending to the lower concentrations, i.e., from 10^− 15^ to 10^− 21^ g/ml (40–70% of responsiveness). Thus, the reactive concentration range was as broad as for B 10^− 5^ g/ml but with lower response rates. From conditioning at 10^− 19^ g/ml, pup responsiveness became more and more narrow, restricted to the lowest concentrations, and almost exclusively displayed only to the learning concentration or to adjacent, very similar concentration levels. Thus, after conditioning at 10^− 19^ and 10^− 20^ g/ml, pups responded to B from 10^− 18^ to 10^− 21^ (40–70%) and at 10^− 17, −19, −21^ g/ml (40–65%), respectively. After conditioning at 10^− 21^, they responded only to B 10^− 20^ and 10^− 21^ g/ml (64–76%). Finally, after conditioning at 10^− 22^ g/ml, no pups responded to B 10^− 22^ g/ml (Fig. 1B) or to any of the other concentrations tested (from 10^− 18^ to 10^− 23^ g/ml), demonstrating that they could no longer learn odorant B from 10^− 22^ g/ml, and attesting that the learning concentration threshold for B is ≤ 10^− 21^ and > 10^− 22^ g/ml.

In summary, for both odorants A and B, the pups were able to learn the CS over a wide range of concentrations - with certain limits -, and the learning concentration always induced one of the highest levels of responsiveness. In addition, two main results emerged that were similar for both odorants. First, for the highest concentrations of learning, i.e., 10^− 5^ g/ml for B and 10^− 5^ and 10^− 10^ g/ml for A, behavioural responsiveness showed the most extended generalization, with very low detection/recognition thresholds. Second, for the lowest learning concentrations, the active concentration range shifted to lower concentrations and became narrower around the learning concentration. Thus, the results indicate that the newborn rabbits learned a concentration-dependent odour quality for each odorant, meaning that the learning concentration played a critical role in the newborns’ recall and post-conditioning perceptual abilities.

### Responsiveness to extremely low levels of concentration and order effect

The responsiveness of newborn rabbits to the lowest concentrations tested (below 10^− 20^ g/ml) raised questions about the functioning of olfaction, i.e., how molecules can sufficiently enter into the nares and interact with receptors to generate perception when there are so few of them (see General discussion). Therefore, we wanted to ensure that this responsiveness was not the result of a methodological factor. Indeed, the order in which different stimuli are tested can theoretically affect how organisms respond to them. Here, testing the same rabbits at several different concentrations in succession could generate responses linked to an order effect. For example, exposure to a high concentration first could result in a response to a very low concentration later on. We took several precautions to avoid this, such as presenting the concentrations in a different order for each newborn from a same litter, leaving a sufficient time interval between each presentation to the same pup, and conducting blind testing (see the Methods section for details). However, to ensure that there was definitely no order effect, we have tested 39 other newborn rabbits (from 6 litters) as follows: (1) 14 pups were conditioned exactly as before (i.e., by single pairing with MP at 10^− 5^ g/ml) to odorant A at 10^− 21^ g/ml; (2) 15 pups were conditioned to odorant A at 10^− 22^ g/ml; (3) 10 pups were conditioned to odorant A at 10^− 23^ g/ml (negative control group). Twenty-four hours later, each of the three groups was tested at the conditioning concentration only, followed by testing at MP 10^− 5^ g/ml. If there were an order effect, the levels of responsiveness to the odorant A (i.e., the proportions of pup responding) would differ from those observed in Fig. 2. Conversely, if the pups’ responsiveness remained the same, this would demonstrate that successive presentations do not affect them.

As a result, 93%, 87% and 0% of the pups responded to A 10^− 21^, 10^− 22^ and 10^− 23^ g/ml, respectively, 24 h after conditioning. These results were not significantly different to those observed after successive testing of different concentrations of A (100%, 85%, 0%, respectively in Fig. 2; χ² < 0.5, *p* > 0.05). At the same time, 100% of the pups responded to MP.

Thus, the responsiveness displayed by the rabbit pups in our experiments at extremely low concentrations did not appear to be due to an order effect.

## Discussion

The aim of the present study was to investigate whether, when newborn rabbits learn a new odorant, the concentration at which the odorant is learned influenced the qualitative generalization of the learned stimulus, i.e., the range of concentrations to which the pups subsequently responded; and, if so, whether this differs as a function of the concentration level (high or low) at the time of learning. We evaluated this by harnessing the powerful ability of newborn rabbits to learn novel odorants through direct pairing with the naturally relevant mammary pheromone (associated with milk intake and survival in this species). The results largely extend our initial observations^29^, by applying the same procedures but with two odorants and over a much wider range of concentrations.

Under our experimental conditions, newborn rabbits exhibited remarkable sensory and cognitive abilities (which we discuss in detail below). Under these rigorously controlled conditions (see the Methods section), the conditioned odours elicited neonatal responsiveness at certain concentrations but clearly not at others, a result consistently obtained with two odorants of opposite volatility. This indicates, firstly, that our procedures reveal not only the remarkable abilities of rabbit pups, but also the existence of some limitations in their ability to detect and integrate novel stimuli; and secondly that the responsiveness of the pups was due solely to the presented stimuli and not to contextual artefacts that might interfere with them.

At the highest concentrations, exceeding 10^− 5^ g/ml, newborn rabbits were unable to learn the odorants A (highly volatile) and B (weakly volatile) in our experimental conditions (i.e. with MP used as the unconditioned stimulus at 10^− 5^g/ml in mixture with A or B). For A at 10^− 2^ and 10^− 3^ g/ml, behavioural non-reactivity of naive (i.e., unconditioned) newborns to the A + MP mixture revealed that odorant A masked the perception of the MP (A was then about 6000 and 600 times more concentrated than the MP at 10^− 5^ g/ml). This masking prevented association and learning^36,37^. In contrast, for odorant B at 10^− 2^ or 10^− 3^ g/ml, naive newborn rabbits responded to B + MP, showing that odorant B did not mask the perception of the MP (given its low volatility, B was 2000 and 200 times less concentrated than MP). Nevertheless, learning of B failed to take place. This lack of learning may be due to the amount of alcohol required to make B soluble (B was poorly soluble at these concentrations and required 1 and 0.1% of alcohol at 10^− 2^ and 10^− 3^ g/ml). The alcohol may irritate the pups and/or produce an unpleasant sensation, thus interfering with the learning of odorant B.

From 10^− 5^ g/ml and below, we discuss the responsiveness of newborn rabbits in four points.

First, rabbit pups learned the odorants along a particularly wide range of concentrations. Indeed, rabbit pups learned odorant A down to 10^–22^ g/ml, and odorant B down to 10^–21^ g/ml. These learnable concentrations are *de facto* the lowest concentrations that could have been perceived on the day of co-exposure with the MP. They thus correspond to the spontaneous detection thresholds of odorants A and B in our conditions. Knowing that these results hold both for a naturally high (A) and low volatile odorant (B), one may assume that they can be generalised to most odorants. This highlights the capability of newborn rabbits to detect and learn novel stimuli in their environment even at impressively low concentrations, at least in association with the major biological signal spontaneously provided by the MP in this species. It is also important to note that the MP imparts biological significance on odorants A and B at concentrations at which it is not itself spontaneously active as a behavioural releaser (active range of MP as releaser in newborn rabbits: 2.5 × 10^–5^ to 2.5 × 10^–9^ g/ml [38]).

Second, the pups showed the widest continuous range of responses for the highest learnable concentrations of CS, i.e., 10^− 5^/10^− 10^ g/ml for A and 10^− 5^ g/ml for B. Indeed, for these learning concentrations, the pups were able to respond after conditioning to odorant A down to 10^− 21^ g/ml and after conditioning to odorant B down to 10^− 19^ g/ml, i.e., up to 17 and 15 log-units lower than the concentration at learning. These results highlight that even early in life, animals can generalise a learned stimulus quality when it is subject to large quantitative variations, ensuring the perception of an odour quality continuum; however, this process has certain limitations, probably physiological and cognitive, as can be seen from our results. In animals - including humans - generalisation is a fundamental feature of odour-guided adaptive behaviours^38–41^ and most researchers agree that it constitutes a priority in the general functioning of the olfactory system^42–46^. To meet this priority, our results suggest that, spontaneously, the rabbit olfactory system commonly operates within a concentration range limited to lower concentrations of 10^− 10^ for A and 10^− 15^ g/ml for B. Above this limit, i.e. over a range of concentrations encompassing intermediate and high concentrations, odour identity might be spontaneously invariant^46^. However, the MP-induced learning could push back this limit and allow much wider generalisation. This would be particularly useful in fasted newborns, as here, i.e., in pups which are in a state of maximum wakefulness^47,48^.

Third, at learning concentrations lower than 10^− 10^ and 10^− 15^ g/ml for odorants A and B, the pups did not respond to the highest concentrations during behavioural testing, revealing a disruption in the perceived quality of the stimulus. From this disruption, the distribution of behavioural responsiveness indicates that new quality/qualities would emerge, inextricably dependent on intensity. This could create a generalisation gradient around the learned concentration, at which the qualitative attributes of the stimulus are optimally recognised. Below and above the learned concentration, the proportion of responding pups decreased, most likely in proportion to the perceived ‘dissimilarity’ in stimulus quality^49,50^. Thus, if a pup was conditioned to a high concentration, it generalized to all concentrations down to a very low one. A question might then be: why doesn’t it generalize to the same low concentrations when conditioned to a slightly lower concentration? At high concentrations, learning process probably implies that almost all the receptor neurons sensitive to the odorant were recruited and thus stimulated^51^. In contrast, at low concentrations, learning probably involved stimulation of a much smaller subset of receptor neurons (only the most sensitive)^52^. This hypothesis could explain the observed asymmetry in generalization. Indeed, at high concentrations of CS, the peripheral activity elicited by lower concentrations during post-conditioning tests would correspond to only a fraction of the activity expressed and encoded during learning; however, from this fraction, the phenomenon of ‘pattern completion recognition’^1,53^ could come into play, allowing the animal to recognize the stimulus as similar to CS. Conversely, at low concentrations of CS, the peripheral activity generated by higher concentrations has never been induced before, and never memorized, so the phenomenon of pattern extension from the encoded fraction cannot occur. In any case, the results showed that, at low concentrations, the pups then learn a quality more dependent on the concentration of the odorant - change the concentration and the quality changes such that during retention test there is no congruence, and no response. So there would be a quality/intensity tuning related to the concentration. The existence of such a tuning may have important implications for the design of learning protocols aimed at detecting odorants in natural olfactory scenes; the protocols should be adapted to the expected target concentrations^54^. This concept may open new insights on the coding of olfactory quality in general and stimulate further work to explore the neurophysiological basis of this coding.

Fourth, the lower the CS concentration at learning, the lower the detection/recognition threshold. Thus, when odorant A was learned at concentrations from 10^− 20^ to 10^− 22^ g/ml, and odorant B from 10^− 19^ to 10^− 21^ g/ml, the after-learning detection threshold was 10^− 24^ g/ml and 10^− 21^ g/ml, respectively. Importantly, these concentrations were not learnable by rabbit pups (i.e., cannot be used as CS) but became perceived after MP-induced conditioning. Thus, such conditioning not only increased the operating range of the olfactory system and thus qualitative generalisation, but also can slightly increase the olfactory detection performance of rabbit pups. The peripheral plasticity may explain, at least in part, this gain in sensitivity^29^. How do the detection thresholds we have identified in newborn rabbits compare with the lowest thresholds reported in the mammalian literature? We propose here a comparison, with the caveat that the experimental conditions are not identical (e.g., not at the same developmental stage of organisms, not with the same odours, the same experimental/learning procedures, etc.). For instance, after conditioning, adult mice can detect bourgeonal at 10^− 4^ ppt^55^ and isoamyl acetate at or below 1 ppt^56^. In adult dogs, the reported post-conditioning behavioural threshold for amyl acetate was 1–2 ppt^57,58^. In newborn rabbits, using odorant A, whose volatility properties are closest to those of isoamyl acetate and bourgeonal, the post-learning detection threshold was 10^− 24^ g/ml, equivalent to approximately 10^− 8^ ppt (g/g) (Table 1). Thus, newborn rabbits would have a sensitivity 10^4^ to 10^8^ times greater than those of adult mice and dogs. This may be because newborn mammals need to quickly learn about their environment, and that their developing brains are particularly well suited to forming associations easily. As mentioned above, this difference may also be partly due to our experimental conditions. Indeed, we worked with rabbit pups: (a) in a learning paradigm using an unconditioned stimulus, the MP, which is of very high spontaneous biological value, essential for newborn rabbits’ survival; (b) the same stimulus (MP) was used to give a conditioned stimulus the power to release the behavioural response initially elicited by the MP alone; (c) we compared the responsiveness to the CS after the pups had learned it at different concentrations. In comparison, studies in mice and dogs did not use a pheromone as an unconditioned stimulus and tested the consequences of learning a CS at only one concentration during conditioning.

**Table 1:** Concentrations of stimuli in aqueous phase. Odorant A: Ethyl isobutyrate (CAS# 97–62–1), MW (molecular weight) = 116.16 g. Odorant B: Ethyl maltol (CAS# 4940–11–8), MW = 140.14 g. Concentration in ppt (part per trillion g/g) = C _mol/l_ x MW_g_ x 10^12^.

Odorant	Concentration (g/ml)	Concentration (mol/l)	Concentration (ppt)
A^− 2^	1 × 10^− 2^	8.61 × 10^− 2^	1 × 10^14^
A^− 3^	1 × 10^− 3^	8.61 × 10^− 3^	1 × 10^13^
A^− 5^	1 × 10^− 5^	8.61 × 10^− 5^	1 × 10^11^
A^− 10^	1 × 10^− 10^	8.61 × 10^− 10^	1 × 10^6^
A^− 15^	1 × 10^− 15^	8.61 × 10^− 15^	10
A^− 17^	1 × 10^− 17^	8.61 × 10^− 17^	1 × 10^− 1^
A^− 19^	1 × 10^− 19^	8.61 × 10^− 19^	1 × 10^− 3^
A^− 20^	1 × 10^− 20^	8.61 × 10^− 20^	1 × 10^− 4^
A^− 21^	1 × 10^− 21^	8.61 × 10^− 21^	1 × 10^− 5^
A^− 22^	1 × 10^− 22^	8.61 × 10^− 22^	1 × 10^− 6^
A^− 23^	1 × 10^− 23^	8.61 × 10^− 23^	1 × 10^− 7^
A^− 24^	1 × 10^− 24^	8.61 × 10^− 24^	1 × 10^− 8^
B^− 2^	1 × 10^− 2^	7.14 × 10^− 2^	1 × 10^14^
B^− 3^	1 × 10^− 3^	7.14 × 10^− 3^	1 × 10^13^
B^− 5^	1 × 10^− 5^	7.14 × 10^− 5^	1 × 10^11^
B^− 10^	1 × 10^− 10^	7.14 × 10^− 10^	1 × 10^6^
B^− 15^	1 × 10^− 15^	7.14 × 10^− 15^	10
B^− 17^	1 × 10^− 17^	7.14 × 10^− 17^	1 × 10^− 1^
B^− 19^	1 × 10^− 19^	7.14 × 10^− 19^	1 × 10^− 3^
B^− 20^	1 × 10^− 20^	7.14 × 10^− 20^	1 × 10^− 4^
B^− 21^	1 × 10^− 21^	7.14 × 10^− 21^	1 × 10^− 5^
B^− 22^	1 × 10^− 22^	7.14 × 10^− 22^	1 × 10^− 6^
B^− 23^	1 × 10^− 23^	7.14 × 10^− 23^	1 × 10^− 7^

Together, the lowest learnable and post-learning detectable concentrations observed in our study demonstrate that rabbit pups have a very high odour sensitivity. Although the results were unexpected at low concentrations, their remarkable consistency and reproducibility, combined with our methodological controls and validations, give them scientific validity. However, we are fully aware of the extremely low theoretical concentrations of A and B sometimes used, particularly below 10^− 20^ g/ml. Given the current state of knowledge regarding olfaction, we are not yet able to explain how a very small number of molecules can generate the detection and perception of a signal in rabbit pups at such low concentrations. To facilitate comparison with some values of olfactory sensitivity reported in the literature, Table 1 shows the liquid-phase concentrations of A and B expressed in mol/L and ppt (g/g). It should be noted that these values are estimated based on serial dilution calculations, and that the actual concentrations of the stimuli in the gaseous phase are unknown. To date, to the best of our knowledge, no instrument is sensitive enough to measure such concentrations directly.

Although the literature does not report such concentration values by name, olfactory sensitivities of this order may be involved in the behaviour of other aerial-breathing animals in nature. Indeed, the lowest concentrations used here may correspond to the conditions that animals encounter in open spaces when detecting or following either scent trails, i.e., stimuli that have become biologically relevant through experience (learning), or spontaneously relevant stimuli such as pheromones^59^. For example, after learning artificial odours under experimental conditions, dogs^60,61^ or butterflies in the wild^62^ can follow olfactory trails/pheromonal plumes for several kilometres. In nature, thousands of m^3^ of air dilute olfactory trails, even if one supposes that the heaviest molecules will be deposited on the ground or distributed according to an organized ‘moving plume’. As an illustration, *Bombyx Mori* males can detect as few as 2.8 × 10^− 22^ mol/l in the gradient of molecules of bombykol released by females, which constitute the pheromone plume; only one pheromone molecule is then needed to elicit a nerve impulse in olfactory sensory neurons^11^. Remarkably, the capabilities observed in newborn rabbits during our experiments are at the same order as those observed in *Bombyx mori*, with rabbit pups’ detection thresholds of 8.61 × 10^− 24^ mol/l for A and 7.14 × 10^− 21^ mol/l for B (Table 1). Although this comparison involves an insect and a mammal, as well as direct responsiveness to a pheromone versus to an odorant learned through pheromone-induced conditioning, the shared factor of pheromone signaling bridges this phylogenetic gap: it directs mate-searching in silk moths and food-searching in newborn rabbits - two behaviors critical for survival. In both cases, pheromones form the basis of intense behavioral motivations that probably prompt the olfactory system and other sensory systems to function under extreme conditions.

In conclusion, this study shows that mammary pheromone-induced learning in newborn rabbits produces a remarkable sensitivity to the learned odorant, a sensitivity that varies according to the concentration at which the odorant was learned. Notably, these effects are observed whether the conditioned stimulus is an odorant of high or low volatility. From an ecological perspective, the remarkable ability of newborn rabbits to learn and respond to new information, depending on the chemical context in which learning occurs, is adaptive. In the wild, the nature and intensity of the odorants carried and emitted by rabbit mothers can vary between females, probably depending on factors such as age, physiological and social status, and diet. It is therefore crucial for newborns to be able to adapt to this variability in order to suck efficiently when the mother visits the nest (for only five minutes per day), and to learn about their mother’s environment and that of their social group, which they will encounter when they are old enough to leave the nest. In terms of generalisation, the results show that if newborns are exposed to relatively high stimulus concentrations during learning, they subsequently demonstrate broad qualitative generalisation with respect to the conditioned stimulus; conversely, if they are exposed to low concentrations during learning, they subsequently respond only to concentrations closely tuned to the learned concentration. In the first case, this may be a way for newborns to learn information that is widespread in their environment and that it will be useful for them to recognise later on, even when it undergoes variations in chemical intensity (e.g., odors from familiar individuals in their own or other social groups, or from familiar food, which may vary according to the seasons). In the second case, it may facilitate the identification/recognition of highly specific information that must be clearly distinguished from other information (e.g., odors allowing the recognition of the mother from other lactating females, or the perception of non-social signals that are crucial for survival, such as danger cues).

Taken together, the results confirm that newborn rabbits are a particularly valuable model for exploring the functioning of olfaction, including in conditions of concentration that have rarely or never been studied before in aerial and aquatic animals^10,11,63^. Studying their olfactory capacities will allow researchers to further uncover the fascinating perceptual and biological abilities displayed by animals, particularly during the early stages of life.

## Methods

### Ethics

This work is reported in accordance with ARRIVE guidelines and strictly followed the local, institutional and French national rules regarding the care and experimental uses of the animals. Thus, all experiments were conducted in accordance with ethical guidelines enforced by French Law (French Ministries of Agriculture, and of Research & Technology) and approved by the Ethical Committees for Animal Experimentation of the University of Lyon 1 (CEEA-42 and − 55) and the French Ministry of Higher Education and Research (no. APAFIS #27874–2020110416356847 v2).

### Animals

New-Zealand rabbits (Charles River strain, France) originated from the breeding colony of the Centre de Recherche en Neurosciences de Lyon. 12 adult males and 40 adult females *Oryctolagus cuniculus* (Charles River strain, L’Arbresle, France) were kept in individual cages under a constant 12:12 light-dark cycle (light on at 7:00 a.m.), with ambient air temperature maintained at 21–22 ◦C. Water and pelleted food (Lapin Elevage 110, Safe, France) were provided *ad libitum*. The study, which lasted over two years (19 experimental sessions were conducted), used two consecutive parent herds, one before and one after the peak of COVID pandemic in France. Two days before the expected day of parturition (day of delivery was considered postnatal day 0; d0), a nest box (0.39 × 0.25 × 0.32 m) was fixed to the cages of pregnant females. To even out pup-female interaction, females’ access to the nest was allowed for 15 min per day at 11:30 a.m. (this procedure allowed mimicking the short daily nursing displayed by rabbit females^64^. We used 387 pups of d1-4 from 67 litters.

### Odorants

The odorants consisted of 2-methylbut-2-enal (the Mammary Pheromone, MP, CAS# 497-03-0)^19–29^, ethyl isobutyrate (odorant A; CAS# 97-62-1) and ethyl maltol (odorant B; CAS# 4940-11-8) (purity of the odorants ≥ 99%)^26,29,66^. The odorants A and B had very high and low volatility properties, respectively, characterized by a factor of volatility approximately 167 000 higher for A compared to B (vapour pressure saturation: 3.23 × 10^3^ Pa for A versus 1.93 × 10^− 2^ Pa for B). To give an idea in the gas phase, the ratio of A and B can be estimated using Henry’s law constants (as is standard practice in olfaction): A is approximately 1.3 × 10⁶ times more concentrated than B, at all dilution levels. Thus, the odorants A and B were representative of the opposite poles of the various volatilities of environmental chemical components, enabling us to generalise our observations.

The MP allowed us to induce learning of odorants A or B through associative conditioning (see below, the odour conditioning section). The MP served as the unconditioned stimulus and was used at 10^− 5^ g/ml, a concentration known to be highly efficient in promoting conditioning^24,25,29^, while the A and B odorants served as the conditioned stimuli (CS). For A and B, the main set of concentrations mixed with the MP was 10^− 5^, 10^− 10^, 10^− 15^, 10^− 17^, 10^− 19^, 10^− 20^, 10^− 21^, 10^− 22^ g/ml, and 10^− 23^ g/ml for A only. In addition, in separate tests and for both odorants, two higher concentrations were also mixed with MP, 10^− 2^ and 10^− 3^ g/ml. During behavioural testing, MP was also used at 10^− 5^ g/ml and single odorants A and B were used at concentrations varying from 10^− 5^ to 10^− 25^ g/ml (odorant A), and from 10^− 5^ to 10^− 22^ g/ml (odorant B) were also used (see below).

All the odorants were purchased from Sigma-Aldrich (Saint-Quantin Fallavier, France). The final solutions were obtained from stock solutions at 10^− 2^ g/ml prepared in 0.1% of ethanol (anhydrous, Labelians, Nemours, France) and distilled water; these stock solutions were renewed several times a year (in new vials). Then, successive dilutions were prepared in distilled water only, following dilution steps of 100, excepted for 10^− 3^ and 10^− 15^ g/ml, which were prepared following dilution steps of 10. For each experimental session, we prepared new series of dilutions in new vials. All preparations were made in a dedicated odorant storage and preparation room in our laboratory, under a hood.

### Odour conditioning

Conditioning sessions were run on days 1, 2 or 3 in an experimental room adjacent to the breeding room. These two rooms were isolated, each closed by a door, with no windows on the outside so no possible contamination from the outside. They were completely separate from the room where odour solutions were prepared (not on the same floor of our building). A ventilation system ensured constant and optimal air renewal in the two rooms. The pups were transferred from the breeding to the experimental room in groups of 5 (from the same litter) into a box lined with nest materials and maintained at room temperature (experimenters wore lab coats, hand gloves and no perfume on the days of the experiments). The MP-induced conditioning consisted of a single, brief and simultaneous exposure both to the MP and to the conditioned stimulus: 8 ml of the MP-A or MP-B mixtures were pipetted onto a cotton pad (19 × 14 cm, 100% cotton), then held 2 cm above the pups for 5 min. This exposure is known to induce very rapid learning of the stimulus paired with the MP^24–29^. The conditioning session took place 1 h before the daily nursing (10:30 a.m.), to standardise the pups’ motivational states and limit the impact of satiation on responses^47^. Two minutes after the end of the conditioning, the pups were individually marked with weakly odorous ink and returned to their nest (it was indeed important to be able to identify them individually until the next day - the day of behavioural test - because of the test procedure; see the section immediately below). The box containing the pups was rinsed with alcohol and distilled water after each conditioning session. Around 20 pups from 4 different litters were conditioned to the same concentration of the conditioned stimulus in order to control for individual differences; each of these groups were then split into two subgroups of 9 to 11 newborns (from 2 litters) for behavioural testing (see below).

### Behavioural assay

Behavioural testing took place on days 2, 3 or 4, i.e., 24 h after phase 1 (conditioning), in the same experimental room as for conditioning (by experimenters again wearing lab coats, hand gloves and no perfume). It was also run 1 h before daily nursing to limit the impact of satiation on pups’ motivation and responsiveness^47^. The assay consisted of an oral activation test during which a pup was immobilized in one gloved hand of the experimenter, its head being left to move freely. Each CS (odorant A or odorant B) at different concentrations were presented for 10 s, as was the MP as a control at a constant concentration (10^− 5^ g/ml), using a glass rod held 0.5 cm in front of the nares, immediately after immersion in the diluted solution^18–29^. Each stimulus concentration had its own glass rod, which was rinsed/dried between animals and cleaned daily with alcohol.

A test was positive when the CS elicited the stereotyped on/off response normally elicited by the MP, i.e., head-searching movements (vigorous, low amplitude horizontal and vertical scanning movements displayed after stretching towards the rod) usually followed by grasping movements (oral seizing of the rod extremity). Non-responding pups displayed no response except active sniffing. Pups were tested 5 by 5, so that the two subgroups of *n* = 10 allowed us to cover the range of concentrations evaluated for odorant A, as for odorant B. Each pup participated in only one experiment and was tested at a maximum of 5–6 concentrations of the conditioned stimulus (and to MP at the end) to prevent fatigue or habituation. Successive concentrations were tested blind to the experimenters. In practice, two experimenters were present at each test session and the one who actually performed the test with the rabbits (experimenter 1) did not know the concentrations he was testing. These concentrations were provided to him by experimenter 2 who held out the relevant vial without experimenter 1 being able to see the concentration indicated on it. The test consisted of the presentation of a first stimulus to a pup, its reintroduction into the box, then the stimulation of another pup with a second stimulus, its reintroduction into the box, and so on with all pups and all stimuli of a given series by respecting an inter-trial interval of 60 s. The order of presentation of the concentrations was strategically interspersed from one newborn to the next to prevent habituation/sensitization/extinction, except for the penultimate and the last stimulation, which were always the same, i.e. the CS at the learning concentration then the MP used as a control.

When a pup responded to a stimulus, it usually made direct nasal contact with the end of the rod, often even grasping it in mouth, so its nose was gently dried with absorbent paper before the pup was returned to the box and exposed to further stimulation. The pups were immediately reintroduced to the nest after the end of their testing session, before a new group of 5 pups was tested in turn.

### Statistical analysis

Pup responding frequencies were compared using the χ² Pearson test when the groups or subgroups were independent (i.e., distinct groups/subgroups tested for their response to the same stimulus) or Cochran’s Q test when the groups/subgroups were dependent (i.e., pups from the same group/subgroup tested for their response to several stimuli). When the Cochran’s Q test was statistically significant, proportions of responding pups were compared 2 × 2 by the χ² McNemar test. Statistical analyses were performed using XLSTAT software (Microsoft, Redmond, USA). Degrees of freedom are indicated when > 1. Effects were considered significant at *p* < 0.05.

## Supplementary Information

Below is the link to the electronic supplementary material.

Supplementary Material 1

Supplementary Material 2

## Data Availability

The full raw dataset is provided in an additional file.
